# High diversity of *Salmonella* spp. from children with diarrhea, food, and environmental sources in Kilimanjaro – Tanzania: one health approach

**DOI:** 10.3389/fmicb.2023.1277019

**Published:** 2024-01-03

**Authors:** Ephrasia A. Hugho, Happiness H. Kumburu, Kate Thomas, AbdulHamid S. Lukambagire, Boaz Wadugu, Nelson Amani, Grace Kinabo, Tine Hald, Blandina T. Mmbaga

**Affiliations:** ^1^Biotechnology Research Laboratory, Kilimanjaro Clinical Research Institute, Moshi, Kilimanjaro, Tanzania; ^2^Institute of Public Health, Kilimanjaro Christian Medical University College, Moshi, Tanzania; ^3^Department of Biochemistry, Kilimanjaro Christian Medical University College, Moshi, Tanzania; ^4^Ministry of Primary Industries, New Zealand Food Safety, Wellington, New Zealand; ^5^EcoHealth Alliance, New York, NY, United States; ^6^Department of Pediatrics, Christian Medical Center, Kilimanjaro, Moshi, Tanzania; ^7^Faculty of Medicine, Kilimanjaro Christian Medical University College, Moshi, Tanzania; ^8^Research Group for Genomic Epidemiology, Technical University of Denmark, Lyngby, Denmark

**Keywords:** *Salmonella* spp., diarrhea, food, water, one-health, whole genome sequencing

## Abstract

*Salmonella* is one of the most frequent causes of diarrhea globally. This study used a One Health approach to identify *Salmonella* species in children admitted with diarrhea and tested samples from the cases’ household environment to investigate their genetic similarity using whole genome sequencing. Surveillance of hospitalized diarrhea cases among children under 5 years was conducted in rural and urban Moshi Districts in the Kilimanjaro Region of Tanzania from July 2020 through November 2022. Household visits were conducted for every child case whose parent/caregiver provided consent. Stool samples, water, domestic animal feces, meat, and milk were collected and tested for *Salmonella*. Isolates were sequenced on the Illumina NextSeq platform. Multilocus Sequence Typing and phylogenetic analyses were performed to map the genetic relatedness of the isolates. *Salmonella* was isolated from 72 (6.0%) of 1,191 samples. The prevalence of *Salmonella* in children with diarrhea, domestic animal feces, food, and water was 2.6% (*n* = 8/306), 4.6% (*n* = 8/174), 4.2% (*n* = 16/382), and 17.3% (*n* = 39/225), respectively. Four (1.3%) of the 306 enrolled children had a *Salmonella* positive sample taken from their household. The common sequence types (STs) were ST1208, ST309, ST166, and ST473. *Salmonella* Newport was shared by a case and a raw milk sample taken from the same household. The study revealed a high diversity of *Salmonella* spp., however, we detected a *Salmonella* clone of ST1208 isolated at least from all types of samples. These findings contribute to understanding the epidemiology of *Salmonella* in the region and provide insight into potential control of foodborne diseases through a One Health approach.

## Introduction

*Salmonella* is an important foodborne pathogen identified in food, environmental sources, livestock, and humans (WHO, 2018). The 2017 Global Burden of Disease (GBD) study reported high *Salmonella* infection among under 5 years children, with the highest burden reported in sub-Saharan Africa (SSA) and South Asia (Stanaway et al., 2019). Most salmonellosis cases in humans are food-related (Pires et al., 2015). The onset of symptoms such as fever, headache, stomach cramps, loss of appetite, diarrhea, and vomiting usually occurs within 6 to 72 h of ingestion, and illness usually lasts 2–7 days (WHO, 2018). A review of salmonellosis outbreaks showed that most reported cases in developed countries are linked to the consumption of different types of contaminated food products including broiler meat, pork, egg, and egg products (Popa and Popa, 2021; WHO, 2022). In Africa, few countries have reported salmonellosis outbreaks, one being South Africa (Niehaus et al., 2011; Muvhali et al., 2017; Motladiile et al., 2019). This does not mean that there have not been more *Salmonella* outbreaks in Africa, just that the capacity, resources and prioritization for investigating foodborne outbreaks limits the ability to follow up.

*Salmonella* are capable of surviving for several weeks in a dry environment and for several months in water (Finn et al., 2013; WHO, 2018; Morasi et al., 2022). This hardiness means *Salmonella* cells have the potential to serve as a reservoir for extended periods of time. In order to strengthen the surveillance of *Salmonella*, the role a One Health approach can take is crucial in establishing the prevalence of *Salmonella* in humans, food animals, and environment, and shed light on possible transmission routes (Monte et al., 2021). This approach has revealed the existence of similar serotypes of *Salmonella* in humans and other sources (Kang et al., 2017; Yan et al., 2021). The occurrence of antimicrobial resistant strains of *Salmonella* is of public health concern, particularly through the acquisition of antimicrobial resistant genes through horizontal plasmid and integron transfer (Carroll et al., 2017; Pornsukarom et al., 2018). Reducing foodborne *Salmonella* infection in humans and the associated risks of antimicrobial resistance requires infection control in animals to limit transmission to the environment (Silva et al., 2014).

Technological advancement such as next generation sequencing is currently supporting timely investigations of pathogens of public health importance including *Salmonella. In silico* methods are now used to predict the serotype, however, White-Kauffman-Le Minor scheme remains the gold standard for *Salmonella* serotyping (Bell et al., 2016; Alikhan et al., 2018; Palma et al., 2018). The analysis of WGS data offers a high discriminatory power compared to traditional serotyping methods, which improves typing, traceability and enables the uncovering of novel strains with potential to cause illness (Simon et al., 2017; Pornsukarom et al., 2018; Monte et al., 2021; Yan et al., 2021). Molecular typing of *Salmonella* from different sources is essential for epidemiological investigations, food safety, and public health. Multilocus Sequence Typing (MLST) is a common approach used for microbial typing to assess evolutionary relationships and distribution across hosts and environments (Achtman et al., 2012).

In Tanzania, studies have been conducted on the epidemiology of *Salmonella* species from food animals and food products (Lubote et al., 2014; Crump et al., 2021; Rukambile et al., 2021; Munuo et al., 2022) and surface water sources used for different purposes (Lubote et al., 2014; Lyimo et al., 2016; Anatory and Machang’u, 2018). *Salmonella* infection in Tanzanian children under 5 years old has been conducted in febrile children (Christopher et al., 2013; Msemo et al., 2019). However, there is a scarcity of data reporting foodborne salmonellosis, which is manifested by diarrhea. This study used a One Health approach to identify *Salmonella* in children admitted with diarrhea and test samples from the cases and their household environment to investigate their genetic similarities using conventional microbiology and WGS characterization.

## Methodology

### Study design and timeline

Surveillance of hospitalized diarrhea cases among children under 5 years old was conducted in rural and urban Districts of Moshi, Kilimanjaro, Tanzania, from July 2020 through November 2022. According to the Tanzania population survey of 2012, Kilimanjaro had approximately 191,906 children aged under 5 years [National Bureau of Statistics (NBS) and Office of Chief Government Statistician (OCGS), 2013]. Cases were recruited from eight healthcare facilities in the Moshi Districts where children were admitted with diarrhea. The healthcare facilities included in this study were Kilimanjaro Christian Medical Center, Mawenzi Referral Hospital, Kibosho Hospital, St. Joseph Hospital, TPC Hospital, Pasua Health Center, Kilema Hospital, and Umbwe Dispensary.

### Study population

The study population comprised children aged 0–59 months that were admitted to the study healthcare facilities due to diarrheal illness. The child needed to have lived in the same household as the consenting caretaker for at least 3 months. The study excluded children who contracted diarrhea while hospitalized, those exposed to antibiotics for more than 24 h before enrollment, those with long-term medical illness such as tuberculosis, kidney or liver disease, and any type of enteropathy including cystic fibrosis, Crohn’s disease, celiac disease, ulcerative colitis, or malabsorption syndrome. If consent was given, household visits were conducted for the collection of high-risk exposure sources including domestic animal fecal materials, foods (milk and meat), and water used by the household members for domestic or recreational purposes. Caregivers and siblings were also requested to supply a stool sample for testing.

### Stool collection

One stool specimen per child was collected in a sterile container with transport media (Cary-Blair; Liofilchem Diagnostic–Italy) on the day of enrolment and transported to Kilimanjaro Clinical Research Institute’s Biotechnology Laboratory (KCRI-BL). Briefly, trained research assistants from each healthcare facility were provided with stool containers with spoons and stool collection papers. The child’s caretaker or parent together with research assistants were requested to collect the child’s stool in the stool collection paper. By using the spoon built into the cap of the container, approximately 5 g of the stool was collected and the cap was replaced and tightly closed. Samples were transported in a cold chain to the laboratory and tested within 12 h of collection.

### Collection of household exposure samples

*Water*: Sterile 1 L sample containers were placed underneath a cold-water faucet making sure that the mouth of the bottle did not touch the faucet in order to avoid contamination. Prior to sample collection, the faucet was opened and thoroughly flushed. Flushing was considered complete once the water temperature was stable by feel. The flow of water was adjusted in order to ensure that water did not splash against other surfaces. Only cold water was collected for analysis. To allow for potential pre-chlorination of water sources in urban areas, 0.83 mL of 1% sodium thiosulfate was added to the sample collection containers to neutralize any chlorine in drinking water sources. The same amount of water was collected from other nearby water sources (including rivers, streams, wells, and canals) as reported by the household member. Water from these sources was collected using a sampling poll to hold the sample containers. *Food*: At least 50 g of meat or 50 mL of milk from households, vendors, and shop outlets were collected. *Animal feces*: Samples were collected for all animal species available in a household environment. In the case that multiple animals of a species were present, fresh animal feces/fresh droppings were pooled together to make one sample for that particular group. All samples were labeled appropriately and transported in cool boxes to the KCRI-BL for testing.

### Laboratory procedures

Conventional methods were used to isolate *Salmonella* as previously described by USDA (2019) and Andrews et al. (2018). Briefly, approximately 0.5 g of stool or animal feces samples were pre-enriched with 10 mL of Buffered Peptone Water (BPW; OXOID, Basingstoke, United Kingdom). Milk and meat samples, 25 mL or 25 g were added into 225 mL of BPW in stomacher bags and mixed (Stomacher® 400 Circulator, Seaward, West Sussex, United Kingdom) for 30 s. For water samples, 300 mL of water was filtered using 0.45 μm filter in vacuum pump and transferred immediately to 20 mL of BPW (avoiding filters to become dry). All samples in BPW were incubated at 36 ± 2.0°C for 18 ± 2 h. For the fecal samples, 1 mL of overnight enrichment was aliquoted into 9 mL of Selenite F broth (Lioflchem) and incubated at 36 ± 2.0°C for 18 ± 2 h. For other sample types, 100 μL of each overnight enrichment was aliquoted into 10 mL of Rappaport-Vassiliadis Soy broth (RVS; OXOID) and 1 mL was aliquoted into 10 mL of Muller-Kauffmann tetrathionate-novobiocin broth (MKTTn; OXOID). RVS was incubated at 42 ± 2°C and MKTTn was incubated at 36 ± 2°C overnight. A 10 μL loop full of each selective enrichment was then inoculated onto Xylose Lysine Deoxycholate agar (XLD; OXOID) and Hektoen Enteric agar (HE; OXOID) plates and incubated at 35 ± 2°C for 24 ± 3 h. Typical *Salmonella* colonies were confirmed by Triple Sugar Iron Agar (TSI; OXOID) and Lysine Iron Agar (LIA; OXOID) slants, Urea (OXOID), Indole test and API20E (BioMerieux, France) biochemical test strips according to manufacturer’s instruction.

### Extraction of DNA and WGS

Genomic DNA was extracted from pure overnight cultures grown on blood agar by using Quick-DNA™ Fungal/Bacterial Miniprep Kit (Zymo Research, Irvine, CA, USA) following manufacturer instructions. The quality and quantity of DNA were checked Qubit 2.0 fluorometer (ThermoFisher Scientific, Waltham, MA, USA). Library preparation for sequencing was performed using Illumina DNA Prep Kit (Illumina, Inc. San Diego, CA, USA). Genomic DNA input was fragmented, indexed following limited PCR cycles amplification and PCR cleanup using AMPure XP™ beads (Beckman Coulter, Inc. Brea, CA, USA). Libraries were normalized to ensure equal representation during sequencing. Equal volume of the normalized libraries were pooled, denatured and diluted in hybridization buffer prior to sequencing in the Illumina NextSeq 550 system using a V2 reagent cartridge for the mid-output paired-end 2 × 150 bp protocol (Illumina, San Diego, California 92122, U.S.A).

### Data analysis

Descriptive statistics were performed using STATA version 15 (Stata Corp, College Station, TX, USA). Socio-demographic and clinical characteristics of the children were summarized by frequency and percentages. The prevalence of *Salmonella* was reported as a number of *Salmonella*-positive cultures out of all samples tested and reported as overall prevalence and specific source prevalence. For bioinformatics, raw reads in fastq format were uploaded in Species Finder version 2.0 for species identification based on 16S ribosomal DNA sequence. MLST version 2^1^ was used for sequence typing, and analysis of antimicrobial resistance genes was carried out using ResFinder v.4.1.^2^ Assembled genomes (FASTA format) were used for serotype analysis using SeqSero2^3^ and phylogenetic analysis using CSI Phylogeny version 1.4.^4^ The CSI phylogeny does SNP calling, filter, validation and infers a phylogeny based on the concatenated alignment of high quality SNPs. The default settings used in phylogenetic include 10Xminimum depth at SNP position, 10% minimum relative depth at SNP position, 10 bp minimum distance between SNPs, 30 minimum SNP quality, 25 minimum read mapping quality and minimum z-score of 1.96. *S. enterica* subspecies *salamae* serovar 42:r:- accession number JAAHLF010000001 and *S. enterica* subspecies *enterica* serovar Newport sequence number JAAHJB010000001 from European Nucleotide Archive (ENA) were used as a reference sequence in phylogenetic analysis of *S. enterica* ST1208 and *S. enterica* ST166, respectively. The newick file from CSI phylogeny output were uploaded into FigTree version 1.4.3 for tree visualization and annotation. Raw sequence data have been submitted to the National Center for Biotechnology Information Sequence Read Archive under BioProject ID PRJNA1058431.

### Ethical consideration

This study received approval from the National Institute of Medical Research (NIMR) certificate number NIMR/HQ/R.8a/Vol.IX/3273 and the Kilimanjaro Christian Medical University College, Research Ethics Committee (CRERC), certificate number 2496. Assent for the children and consent for conducting household visits was obtained from the parents or caretakers of the children before enrolment in the study. All participant information was kept confidential.

## Results

### Socio-demographic and clinical characteristics of the study participants

A total of 306 children admitted with diarrhea to one of the eight study healthcare facilities were enrolled in this study. The median age of the children was 13.8 months (IQR 8.4–21.8). Fifty-eight percent were males (*n* = 179) and 59.5% were residing in the Moshi urban area. Vomiting (86.9%) and fever (61.8%) were the most common clinical symptoms reported (Table 1). The majority of children (98%) were taken to seek healthcare by their mothers. The main source of water for daily domestic activities was tap water (>50% of households) and 52% (*n* = 159) of households had animals including cattle, goats, sheep, dogs, pigs, chickens, and ducks in their backyards.

**Table 1:** Demographic and clinical characteristics of the study participants (*N* = 306).

Characteristic	Frequency	Percentage
Child Sex		
Female	127	41.5
Male	179	58.5
Age		
0–11 months	132	43.1
12–23 months	105	34.3
24–59 months	69	22.6
Area of residence		
Rural	182	59.5
Urban	124	40.5
Clinical signs and symptoms		
Vomiting	266	86.9
Fever	189	61.8
Loss of appetite	109	35.6
Coughing	117	38.2
Running nose	108	35.3
Fatigue	45	14.7
Abdominal Pain	22	7.2
Nausea	11	3.6
Flatulence	20	6.5
Headache	1	0.3
Jaundice	1	0.3
Dizziness	1	0.3

### Prevalence of *Salmonella species*

A total of 1,191 samples were collected and tested for the presence or absence of *Salmonella* species. *Salmonella* was isolated from 72 (6.0%) of all samples tested. Of 1,191 samples, 306 were stool samples from children with diarrhea, of which eight samples (2.6%) were confirmed to be *Salmonella* spp. positive by conventional microbiology methods. A total of 179 households were visited and collected between 1 and 10 samples from each household. Overall, 174 fecal samples from different domestic animals, 382 animal derived foods products (meat and milk), and 225 water samples were collected for culture. The prevalence of *Salmonella* spp. from animal feces, foods, and water was 4.6, 4.2, and 17.3%, respectively. Among the other samples obtained, *Salmonella* spp. was isolated from one (1.0%) of 97 caretaker stools but was not isolated from any (0%) of the seven siblings’ stool samples. Table 2 provides information on the samples analyzed. Only 4 (1.3%) children with diarrheal illness were found to have one of the household sample testing *Salmonella* positive in culture as shown in Table 3.

**Table 2:** Clinical, food, and environmental samples analyzed and their culture results (*N* = 1,191).

Sample category	Rural	Urban	Total	Confirmed *Salmonella* positive	% Positive
Human					
Diarrheal stool	182	124	306	8	2.6
Siblings	3	4	7	0	0.0
Caretakers	57	40	97	1	1.0
Animals/poultry					
Cattle	27	3	30	1	3.3
Chicken	63	20	83	5	6.0
Goat	24	5	29	1	3.5
Duck	9	2	11	0	0.0
Pig	12	1	13	1	7.7
Rabbit	1	3	4	0	0.0
Sheep	1	0	1	0	0.0
Dog	0	1	1	0	0.0
Food					
Raw milk	64	49	113	3	2.7
Bovine meat	80	55	135	12	8.9
Pork	12	2	14	1	7.1
Sheep meat	2	1	3	0	0.0
Dairy milk	70	47	117	0	0.0
Water					
Drinking water	144	77	221	39	17.7
Recreational water	4	0	4	0	0.0
Total	757	434	1,191	72	6.1

**Table 3:** Case-household culture positive samples for *Salmonella* (*n* = 4).

Case ID	Sample ID	Location	Source	ST	Serovar name
127	41271121	Urban	Case	1208	II 42:r:-
	41271122	Urban	Caretaker	22	Braenderup
132	41321121	Urban	Case	166	Newport
	41321239	Urban	Raw Milk	166	Newport
176	41761121	Rural	Case	4485	Cerro
	46641231	Rural	Bovine meat	1208	II 42:r:-
351	43511121	Urban	Case	309	Kiambu
	47451231	Urban	Bovine meat	1208	II 42:r:-

### *Salmonella* serotypes and sequence types

Of the 72 *Salmonella* isolates, 56 were sequenced as they had good quality DNA for library preparation. A total of 22 known STs and two previously unknown STs were recorded. The most common serovars isolated were *S. enterica* subsp. *salamae* serovar II 42:r:- (ST 1208, *n* = 21), *S. enterica* subsp. *enterica* serovar Newport (ST166, *n* = 4), *S. enterica* subsp. *enterica* serovar Kiambu (ST309, *n* = 3) and *S. enterica* subsp. *enterica* serovar Oranienburg (ST174, *n* = 3). *S. enterica* subsp. *enterica* serovar Hadar (ST473), *S. enterica* subsp. *enterica* serovar Jangwani (ST3918), *S. enterica* subsp. *enterica* serovar Adelaide (ST2028), *S. enterica* subsp. *enterica* serovar Indiana (ST2040) and *S. enterica* subsp. *enterica* serovar Braenderup (ST22) were all identified twice, with the remaining STs identified once.

Water used by household members for drinking, food preparation, bathing, and washing utensils was commonly contaminated with *Salmonella* spp., and 12 different STs were detected. Eleven isolates of ST1208 were recovered from water, seven of them from tap water in urban areas, and four from rural areas. ST1208 was also recorded from food samples including raw milk (*n* = 2), and bovine meat (*n* = 5) which were obtained from the common selling points reported by parents/caretakers. One *Salmonella* spp. from well water collected in a rural area had an unknown ST, closely related to ST695 or ST2522 (Table 4).

**Table 4:** *Salmonella* sequence types and serovars from human, animal, food and water sources (*N* = 56).

Serovars	ST	Total	Sample source									
			Child case	Caretaker	Bovine meat	Pork	Raw milk	Cattle	Chicken	Goat	Tap water	Well water
II 42:r:-	1,208	21	1		5		2		1		11	1
Kiambu	309	3	1								1	1
Newport	166	4	1				1				1	1
Hadar	473	2									2	
Oranienburg	174	3			1						1	1
Jangwani	3,918	2									1	1
Adelaide	2028	2							1	1		
Indiana	2040	2	1								1	
Braenderup	22	2		1							1	
Agona	13	1							1			
Mango	2,539	1									1	
Neukoelln	288	1										1
Cerro	4,485	1	1									
Durban	2,533	1										1
Herston	6,967	1									1	
Livingstone	2,587	1							1			
II[1],13,23:z29:e,n,x	1,188	1			1							
Umbilo	2014	1			1							
Kentuky	198	1	1									
Give	516	1									1	
Orion	639	1				1						
Typhimurium	19	1									1	
IIIb 50:z52:z53	ND^a^	1						1				
Singapore	ND^b^	1										1
Total		56	6	1	8	1	3	1	4	1	23	8

^a^ND “ST Not Determined” (near ST2861, ST4263, ST4340, ST4649), ^b^ST Not Determined (near ST695 ST2522).

Out of six *Salmonella* isolates from children with diarrhea that were sequenced, four had household samples positive for *S. enterica*. Of these, one carried *Salmonella* of a matching sequence type to that detected in the linked household sample (Case ID 132; ST166) (Table 3). All STs identified, which *Salmonella* serovars they correspond to, and the sample types from which isolates were obtained are shown in Supplementary Table S1.

### Single nucleotide polymorphisms (SNPs)

**Figure 1:**
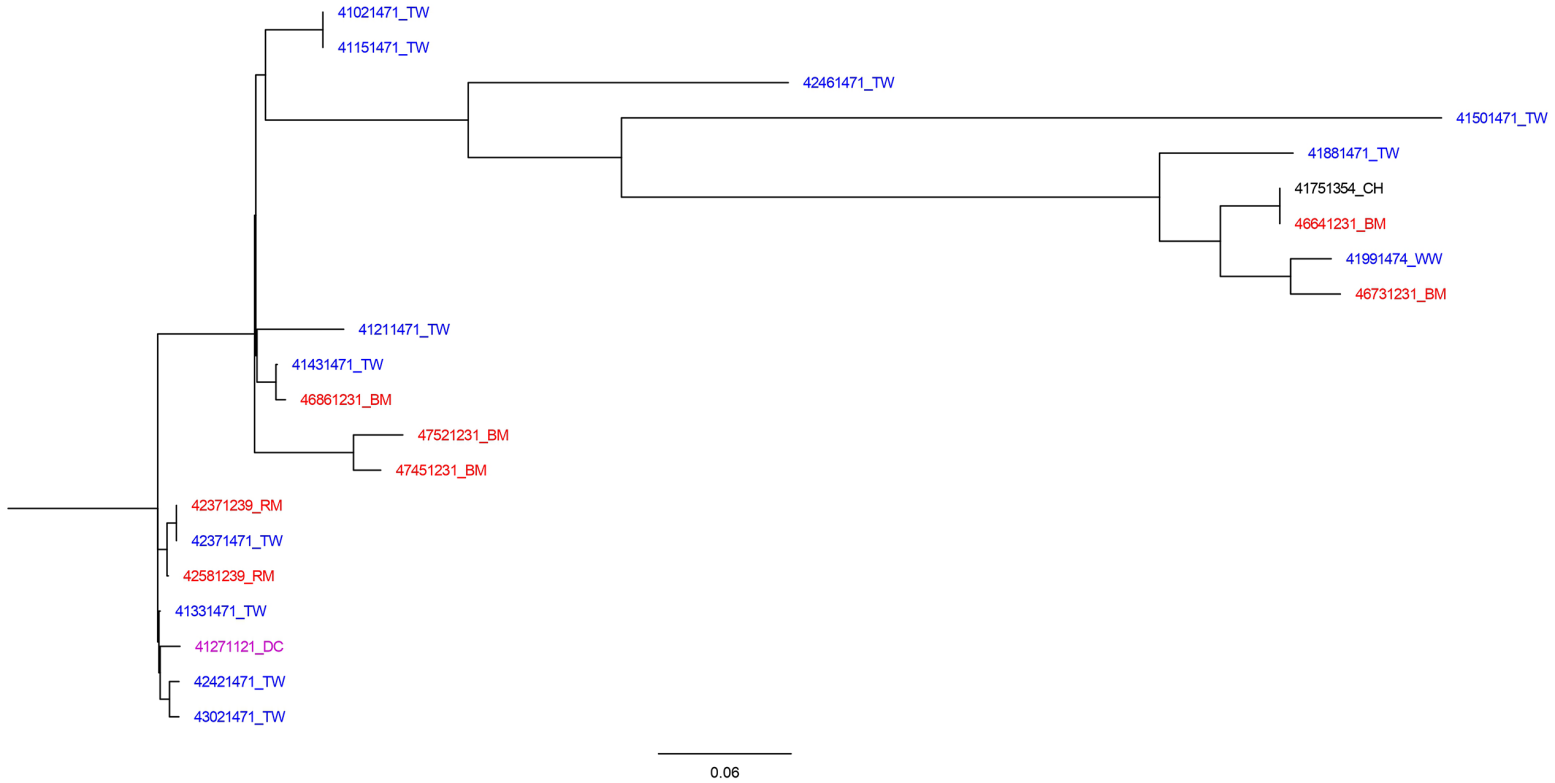
SNP tree of 21 *Salmonella* II 42:r:- genomes constructed using CSI Phylogeny. Blue highlighted isolates were from water sources (TW, tap water; WW, water from wells, springs, or rivers). Red isolates were food sources (BM, bovine meat; RM, raw milk). Black isolates were samples collected from poultry (CH, chicken), and purple represent human source isolates (DC, a child with diarrhea).

A total number of 253 SNP differences were generated by CSI Phylogeny for the most common ST (ST1208) and used to construct a maximum likelihood SNP tree (Figure 1). The minimum and maximum SNP differences were 0 and 111, respectively. The tree was inferred using 21 ST1208 isolates. The tree was observed to have three major branches from the main root. The first branch had two identical isolates recovered from water which shared the common ancestor with isolates identified in water, meat and chicken. This branch subdivided further other small branches of which included identical isolates recovered from meat and chicken fecal sample. Considering the branch length in this group, the tree indicates that there has been more genetic changes over time.

The second branch was composed of isolates from water and meat, and was sub-divided into three other branches. Among these, there were closely related isolates from water and meat with SNP differences of 1 nucleotide. The third branch consisted of cluster of closely related isolates from milk, water, and stool from child with diarrhea with SNP difference of 0–4 nucleotides. The isolates in this group has little genetic changes as indicated by shorter branch lengths.

**Figure 2:**
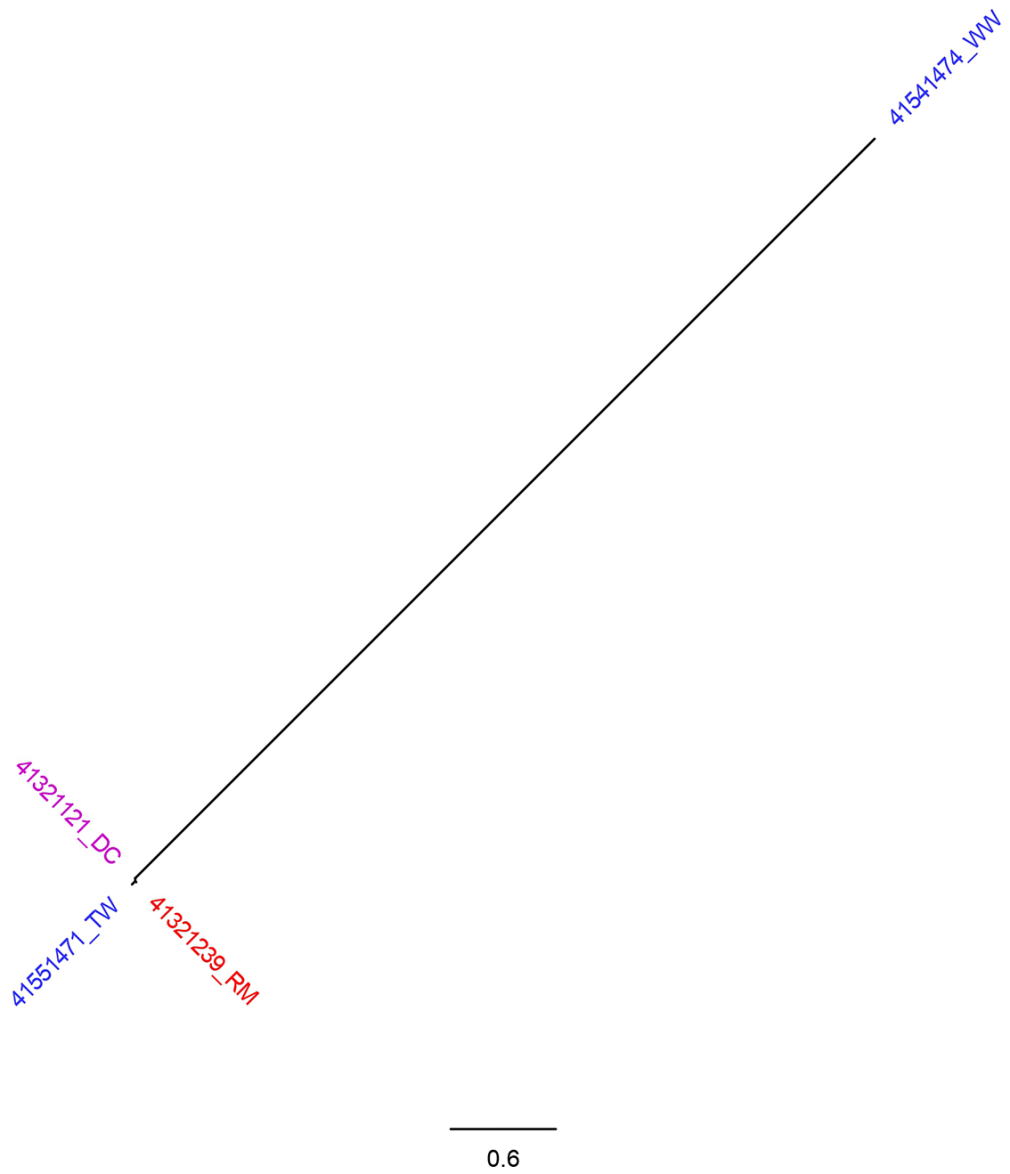
SNP tree of 4 *Salmonella* Newport genomes constructed using CSI Phylogeny. Blue highlighted isolates were from water sources (TW, tap water; WW, water from wells, springs, or rivers). Red isolates were food sources (RM, raw milk) and purple represent human source isolates (DC, a child with diarrhea).

Comparing 4 *S. enterica* subsp. *enterica* serovar Newport (ST166) isolates, a total of 123 SNPs were generated by CSI Phylogeny and used to construct phylogenetic tree. The tree had two branches whereby three of the isolates clustered together (Figure 2). Two of the isolates were identified from child with diarrhea and milk sample from the patients’ house with SNP difference of 3 nucleotides. Tap water collected from the street adjacent to child’s home was closely related with the isolates from the milk sample and child stool with SNP difference of 4 and 5 nucleotides, respectively. Isolate from well water formed its own branch, differing from the other 3 isolates by more than 100 nucleotides.

### Genotypic analysis of *Salmonella* antimicrobial resistance genes (ARGs)

All 56 *Salmonella* isolates were found to have ARGs associated with aminoglycosides. Three (5.4%) isolates additionally carried genes for folate pathway antagonists and tetracycline resistance. One (1.8%) carried genes for fosfomycin resistance, one (1.8%) carried resistance genes to quinolones and one (1.8%) carried genes for resistance to aminocyclitol, aminoglycoside, folate pathway antagonist, quinolone, and tetracycline. Of the six (6) isolates from diarrhea cases, only one (ST198) had genotypic resistance to amikacin (AMI), astromicin, ciprofloxacin (CIP), doxycycline (DOX), fortimicin, gentamicin (GM), nalidixic acid (NA), spectinomycin (SPT), streptomycin (STR), sulfamethoxazole (S), tetracycline (TET) and tobramycin (TOB) antimicrobials. From the food samples, one bovine meat sample had genotypic resistance to AMI, DOX, STR, S, TET, TOB, and TMP. The matched case-household positive cultures were found to have similar AMR patterns (Table 5). More details for each individual isolate are shown in Supplementary Table S2.

**Table 5:** Genotypic analysis of antimicrobial resistance of *S. enterica* by STs.

ST	Serovar	AMR genes	AMR class	AMR resistance*
13	Agona	*aac(6′)-Iaa,fosA7*	Aminoglycoside, fosfomycin	AMI, FOS, TOB
19	Typhimurium	*aac(6′)-Iaa*	Aminoglycoside	AMI, TOB
22	Braenderup	*aac(6′)-Iaa,qnrS13,tet(A)*	Aminoglycoside, quinolone, tetracycline	AMI, CIP, DOX, TET, TOB
166	Newport	*aac(6′)-Iaa*	Aminoglycoside	AMI, TOB
174	Oranienburg	*aac(6′)-Iaa*	Aminoglycoside	AMI, TOB
198	Kentuky	*aac(3)-Id,aac(6′)-Iaa,aadA7,sul1,tet(A)*	Aminocycitol, aminoglycoside, folate pathway antagonist, quinolone, tetracycline	AMI, Astromycin, CIP, DOX, Fortimicin, GM, NA, SPT, STR, S, TET, TOB
288	Neukoelln	*aac(6′)-Iaa*	Aminoglycoside	AMI, TOB
309	Kiambu	*aac(6′)-Iaa*	Aminoglycoside	AMI, TOB
473	Hadar	*aac(6′)-Iaa,aph(3”)Ib,aph(6)-Id,dfrA14,sul2,tet(A)*	Aminoglycoside, folate pathway antagonist, tetracycline	AMI, DOX, STR, S, TET, TOB, TMP
516	Give	*aac(6′)-Iaa*	Aminoglycoside	AMI, TOB
639	Orion	*aac(6′)-Iaa*	Aminoglycoside	AMI, TOB
1,188	II[1],13,23:z29:e,n,x	*aac(6′)-Iaa*	Aminoglycoside	AMI, TOB
1,208	II 42:r:-	*aac(6′)-Iaa*	Aminoglycoside	AMI, TOB
2014	Umbilo	*aac(6′)-Iaa*	Aminoglycoside	AMI, TOB
2028	Adelaide	*aac(6′)-Iaa*	Aminoglycoside	AMI, TOB
2040	Indiana	*aac(6′)-Iaa*	Aminoglycoside	AMI, TOB
2533	Durban	*aac(6′)-Iaa*	Aminoglycoside	AMI, TOB
2539	Mango	*aac(6′)-Iaa*	Aminoglycoside	AMI, TOB
2587	Livingstone	*aac(6′)-Iaa*	Aminoglycoside	AMI, TOB
3918	Jangwani	*aac(6′)-Iaa*	Aminoglycoside	AMI, TOB
4485	Cerro	*aac(6′)-Iaa*	Aminoglycoside	AMI, TOB
6967	Herston	*aac(6′)-Iaa*	Aminoglycoside	AMI, TOB
ND^a^	IIIb 50:z52:z53	*aac(6′)-Iaa*	Aminoglycoside	AMI, TOB
ND^b^	Singapore	*aac(6′)-Iaa*	Aminoglycoside	AMI, TOB

*AMI, Amikacin; TOB, Tobramycin; DOX, Doxycycline; STR, Streptomycin; S, Sulfamethoxazole; TET, Tetracycline; TMP, Trimethoprim; CIP, Ciprofloxacin; GM, Gentamicin; NA, Nalidixic acid; SPT, Spectinomycin; FOS, Fosfomycin. ^a^ST Not Determined (near ST2861, ST4263, ST4340, ST4649). ^b^ST Not Determined (near ST695 ST2522).

## Discussion

This study aimed to identify *Salmonella* in diarrheal children under five years old in rural and urban Moshi districts, Kilimanjaro, Tanzania, and identifying genetic relatedness between isolates from different sources. We report *Salmonella* serovars circulating in the study areas and their genotypic resistance patterns for isolates from humans, food, and the environment. Of particular interest is the *Salmonella* Newport (ST166) isolate that was shared by a case and a raw milk sample taken from the same household.

The prevalence of *Salmonella* among children under five years old seeking healthcare for diarrhea at one of the eight facilities in this study was low, indicating that *Salmonella* are not a common cause of diarrhea in this population. Pathogens such rotavirus, adenovirus, *Shigella* and norovirus have been documented as the leading causes of diarrhea in the same study areas (Hugho et al., 2023). Our findings are in line with similar findings in other studies in Tanzania (Moyo et al., 2011) and Ethiopia and Iraq (Harb et al., 2017; Zelelie et al., 2019), and the reported prevalence of *Salmonella* was low compared to studies conducted in other countries (Azemi et al., 2013; Langendorf et al., 2015). This study identified six distinct STs from isolates obtained in diarrheal samples. This indicates that *Salmonella* infections among the enrolled children in this study occurred sporadically. *S. enterica* subsp. *salamae* serovar II 42:r:- *S. enterica* subsp. *enterica* serovar Kiambu, *S. enterica* subsp. serovar Indiana, *S. enterica* serovar Newport and *S. enterica* subsp. *enterica* serovar Cerro were the serovars identified from the diarrheal cases. Although our study reported low prevalence, it is important to note that non-typhoidal *Salmonella* is a cause of invasive infections, as documented in other countries (Feasey et al., 2012; Stanaway et al., 2019; Ke et al., 2020).

Regarding the distribution of *Salmonella* in different exposure sources, this study found that water used for domestic purposes had the highest prevalence of *Salmonella* followed by foods (meat and milk). The high detection rate of *Salmonella* in water should raise an alarm among Moshi residents as the majority of them drink water directly from the taps. This is due to the myth that the water in Moshi comes from Mount Kilimanjaro and so it is pure. However, this water could be contaminated as it flows from the source to the destination. The detection of *Salmonella* in the samples obtained from the surrounding household environment suggests a high risk of infection transmission to humans, particularly if good hygiene practices are not followed. Most households in Moshi especially in rural areas will have livestock in the backyard. This increases the close proximity between animals and humans hence the high chances of transmission of zoonotic diseases. Previous studies conducted in other regions of Tanzania have reported the presence of *Salmonella* in water, meat, milk, and poultry (Lubote et al., 2014; Lyimo et al., 2016; Anatory and Machang’u, 2018; Munuo et al., 2022).

We found that *S. enterica* subsp. *salamae* serovar II 42:r:- (ST1208) was the common type of *Salmonella* found in the study areas, with frequent isolation from water and bovine meat. *Salmonella* II 42:r:- has been previously reported in East Africa (Crump et al., 2021). The distribution of ST1208 from the phylogenetic analysis revealed the existence of ST1208 clones originating mostly from water sources and food. Although ST1208 was predominant in water used in urban areas, the absence of *Salmonella* from water samples collected from cases’ households makes the source uncertain. However, we found that raw milk and tap water collected from the same household shared ST1208, with a SNP difference of 3 nucleotides. The fact that this genetic strain was found in both raw milk and tap water suggests some form of contamination or shared source between these two media. A difference of 3 nucleotides in the genetic sequence indicates a minor genetic variation, which could potentially be due to mutation, adaptation, or other factors.

Among the Six *Salmonella* positive cases, four had a corresponding household *Salmonella* positive sample. *S. enterica* subsp. *enterica* serovar Newport (ST166) was shared by case and a raw milk sample. The SNP difference between the two isolates was 3 nucleotides indicating that the isolates were closely related but not identical. Based on the parent’s response to the risk exposures, boiled or pasteurized milk reported to have been used by the household members including the ill child, two weeks prior to the onset of the diarrhea. Milk is reported among the potential source of *Salmonella* in many settings (Maichin et al., 2013; Lubote et al., 2014; Silva et al., 2014; ElBaz et al., 2017; Asefa et al., 2023). Additionally, ST166 was detected in tap water from the street adjacent to the residence of the case linked with ST166. The SNP difference among the *S. enterica* subsp. *enterica* serovar Newport from milk and tap water isolates was determined to be 4 nucleotides, while the isolate from child with diarrhea and tap water SNP difference of 5 nucleotides, suggesting a close genetic relationship. This implies that both water and milk present a greater risk of *Salmonella* infection to individuals. Furthermore, *Salmonella* Newport from urban well water had more than 100 SNP differences as compared to the three isolates.

All *Salmonella* isolates had two or more ARGs detected. All isolates carried ARGs for aminoglycosides, with a few of them carrying fosfomycin and quinolones. This indicates a critical concern for infection control from the different sources since the transmission of multidrug resistance isolates to human will associate with high cost of treatment and treatment failures. Similar findings are reported by Crump et al. (2021). Despite our findings being based on genotypic resistance typing, *Salmonella* strains resistant to more than one antimicrobial agent have been reported in studies conducted elsewhere (Madoroba et al., 2016). Waterborne *Salmonella* strains resistant to tetracycline and trimethoprim have been reported in northern Tanzania (Lyimo et al., 2016). Resistance to tetracycline, streptomycin, and nalidixic acid in isolates originating from human feces (*S.* Kentucky) has also been reported elsewhere (Han et al., 2013). *S*. Kentucky isolates resistant to more than one class of antimicrobials has been reported in chicken from Eastern China (Tang et al., 2022). Drugs like amikacin, ciprofloxacin, doxycycline, gentamicin, nalidixic acid, streptomycin, sulfamethoxazole, and tetracycline are routinely used for clinical management of gastroenteritis. The presence of multidrug-resistant strains in water and food samples presents potential threats to human health, particularly if food preparation hygiene is inadequate.

Our study is among few studies reporting *Salmonella* from in humans, food and animals through the One Health approach. Another study reporting the occurrence of *Salmonella* through the One Health approach has been done in Iran. However, different serotypes were isolated in Iran (Badouei et al., 2022). Furthermore, this study was conducted during COVID-19 pandemic. There is limited data on how change in hygiene practices during COVID-19 might have influenced the prevalence of different enteric pathogens including *Salmonella* in under-five children in Tanzania. In areas where there is an active surveillance of *Salmonella* infections, there is evidence that the COVID-19 pandemic-related changes in hygiene practices and health-seeking behaviors may have affected the incidence of enteric pathogens. Finding from studies conducted in Israel and Netherlands found a decrease in the salmonellosis during the COVID-19 pandemic period compared to previous years (Bassal et al., 2021; Mughini-Gras et al., 2021).

One limitation of this study was the inability to obtain leftover food from the ill child’s house to ascertain the source or genetic relatedness of isolates from both the ill child and exposures sources. As a result, food samples like meat and milk were obtained from the places where the household members normally acquired them, as reported by the child’s caretaker or guardian. Another potential limitation could be the administration of antibiotics to children before sampling, however, we excluded those who were reported to have taken antibiotics within 48 h prior to sample collection. Furthermore, the isolates that were not sequenced might have influenced the findings, however, they will be considered in the subsequent analysis of the project data.

## Conclusion

In this study, *Salmonella* spp. were isolated from stool samples from children presenting with diarrhea, water, animal feces, and foods. Different STs were identified, revealing a high diversity of *Salmonella* strains in the study areas, although ST1208 appeared to be commonly found in both human, food, water and poultry. Water used for domestic purposes was highly contaminated with *Salmonella,* followed by animal derived foods (meat and milk). This indicates a high risk of acquiring infection through water and food, hence necessitating the need for community education on the importance of boiling drinking water, milk pasteurization, and adequate cooking. Our findings contribute to understanding the epidemiology of *Salmonella* in the region and provide insight into control of foodborne diseases through a One Health approach.

## Data availability statement

The datasets presented in this study can be found in online repositories. The names of the repository/repositories and accession number(s) can be found in the article/Supplementary material.

## Ethics statement

The studies involving humans were approved by National Health Research Ethics Review Committee. The studies were conducted in accordance with the local legislation and institutional requirements. Written informed consent for participation in this study was provided by the participants’ legal guardians/next of kin.

## Author contributions

EH: Data curation, Formal analysis, Investigation, Methodology, Writing – original draft, Writing – review & editing. HK: Conceptualization, Data curation, Formal analysis, Funding acquisition, Methodology, Supervision, Writing – review & editing. KT: Conceptualization, Funding acquisition, Methodology, Writing – review & editing. AL: Writing – review & editing. BW: Investigation, Writing – review & editing. NA: Investigation, Writing – review & editing. GK: Supervision, Writing – review & editing. TH: Conceptualization, Funding acquisition, Methodology, Supervision, Writing – review & editing. BM: Conceptualization, Funding acquisition, Supervision, Writing – review & editing.

## Footnotes

^1^ https://cge.food.dtu.dk/services/MLST/

^2^ https://cge.food.dtu.dk/services/ResFinder/

^3^ https://github.com/denglab/SeqSero2

^4^ https://cge.food.dtu.dk/services/CSIPhylogeny/
