# Supplementary material for: High diversity of Salmonella spp. from children with diarrhea, food, and environmental sources in Kilimanjaro – Tanzania: one health approach
Source: Front Microbiol. 2024 Jan 3;14:1277019. doi: 10.3389/fmicb.2023.1277019 (PMC10793262; doi:10.3389/fmicb.2023.1277019)
Supplement: Supplementary file 1 [file Table_1.pdf]

Supplementary table S1: Genotypic analysis of antimicrobial resistance of *S. enterica* from human, animal, food and water sources

**Case ID**	**Linked Sample ID**	**MLST**	**Serovar**	**AMR genes**	**AMR Class**	**AMR Resistance***
41021121	41021471	1208	II 42:r:-	*aac(6')-Iaa*	Aminoglycoside	AMI, TOB
41091121	41091351	Unknown	IIIb 50:z52:z53	*aac(6')-Iaa*	Aminoglycoside	AMI, TOB
41131121	41131121	198	Kentuky	*aac(3)-Id,aac(6')-Iaa,aadA7,sul1,tet(A)*	Aminocyclitol, aminoglycoside, folate pathway antagonist, quinolone, tetracycline	AMI, Astromycin, CIP, DOX, Fortimicin, GM, NA, SPT, STR, S, TET, TOB
41151121	41151471	1208	II 42:r:-	*aac(6')-Iaa*	Aminoglycoside	AMI, TOB
41211121	41211471	1208	II 42:r:-	*aac(6')-Iaa*	Aminoglycoside	AMI, TOB
41271121	41271121	1208	II 42:r:-	*aac(6')-Iaa*	Aminoglycoside	AMI, TOB
	41271122	22	Braenderup	*aac(6')-Iaa,qnrS13,tet(A)*	Aminoglycoside, quinolone, tetracycline	AMI, CIP, DOX, TET, TOB
41301121	41301354	13	Agona	*aac(6')-Iaa,fosA7*	Aminoglycoside, fosfomycin	AMI, FOS, TOB
41321121	41321121	166	Newport	*aac(6')-Iaa*	Aminoglycoside	AMI, TOB
	41321239	166	Newport	*aac(6')-Iaa*	Aminoglycoside	AMI, TOB
41331121	41331471	1208	II 42:r:-	*aac(6')-Iaa*	Aminoglycoside	AMI, TOB
41431121	41431471	1208	II 42:r:-	*aac(6')-Iaa*	Aminoglycoside	AMI, TOB
41501121	41501471	1208	II 42:r:-	*aac(6')-Iaa*	Aminoglycoside	AMI, TOB
41541121	41541474	166	Newport	*aac(6')-Iaa*	Aminoglycoside	AMI, TOB
41551121	41551471	166	Newport	*aac(6')-Iaa*	Aminoglycoside	AMI, TOB
41571121	41571471	2539	Mango	*aac(6')-Iaa*	Aminoglycoside	AMI, TOB
41641121	41641471	174	Oranienburg	*aac(6')-Iaa*	Aminoglycoside	AMI, TOB
	41641474	309	Kiambu	*aac(6')-Iaa*	Aminoglycoside	AMI, TOB
41691121	41691471	309	Kiambu	*aac(6')-Iaa*	Aminoglycoside	AMI, TOB
41701121	41701471	2040	Indiana	*aac(6')-Iaa*	Aminoglycoside	AMI, TOB
41711121	41711474	288	Neukoelln	*aac(6')-Iaa*	Aminoglycoside	AMI, TOB
41751121	41751354	1208	II 42:r:-	*aac(6')-Iaa*	Aminoglycoside	AMI, TOB
41761121	41761121	4485	Cerro	*aac(6')-Iaa*	Aminoglycoside	AMI, TOB
41781121	41781471	516	Give	*aac(6')-Iaa*	Aminoglycoside	AMI, TOB
	41781474	Unknown	Singapore	*aac(6')-Iaa*	Aminoglycoside	AMI, TOB
41881121	41881471	1208	II 42:r:-	*aac(6')-Iaa*	Aminoglycoside	AMI, TOB
41931121	41931474	174	Oranienburg	*aac(6')-Iaa*	Aminoglycoside	AMI, TOB

41991121	41991474	1208	II 42:r:-	*aac(6')-Iaa*	Aminoglycoside	AMI, TOB
42091121	42091474	3918	Jangwani	*aac(6')-Iaa*	Aminoglycoside	AMI, TOB
42311121	42311471	2533	Durban	*aac(6')-Iaa*	Aminoglycoside	AMI, TOB
42321121	42321352	2028	Adelaide	*aac(6')-Iaa*	Aminoglycoside	AMI, TOB
	42321354	2028	Adelaide	*aac(6')-Iaa*	Aminoglycoside	AMI, TOB
42371121	42371239	1208	II 42:r:-	*aac(6')-Iaa*	Aminoglycoside	AMI, TOB
	42371471	1208	II 42:r:-	*aac(6')-Iaa*	Aminoglycoside	AMI, TOB
42421121	42421471	1208	II 42:r:-	*aac(6')-Iaa*	Aminoglycoside	AMI, TOB
42431121	42431471	6967	Herston	*aac(6')-Iaa*	Aminoglycoside	AMI, TOB
42461121	42461471	1208	II 42:r:-	*aac(6')-Iaa*	Aminoglycoside	AMI, TOB
42551121	42551121	2040	Indiana	*aac(6')-Iaa*	Aminoglycoside	AMI, TOB
42581121	42581239	1208	II 42:r:-	*aac(6')-Iaa*	Aminoglycoside	AMI, TOB
42631121	42631354	2587	Livingstone	*aac(6')-Iaa*	Aminoglycoside	AMI, TOB
42741121	42741471	22	Braenderup	*aac(6')-Iaa*	Aminoglycoside	AMI, TOB
43021121	43021471	1208	II 42:r:-	*aac(6')-Iaa*	Aminoglycoside	AMI, TOB
43381121	43381471	473	Hadar	*aac(6')-Iaa,aph(3'')-Ib,aph(6)-Id,dfrA14,sul2,tet(A)*	Aminoglycoside, folate pathway antagonist, tetracycline	AMI, DOX, STR, S, TET, TOB, TMP
43411121	43411471	3918	Jangwani	*aac(6')-Iaa*	Aminoglycoside	AMI, TOB
43511121	43511121	309	Kiambu	*aac(6')-Iaa*	Aminoglycoside	AMI, TOB
41251121	46191231	174	Oranienburg	*aac(6')-Iaa*	Aminoglycoside	AMI, TOB
41381121	46301231	1188	II[1],13,23:z29:e,n,x	*aac(6')-Iaa*	Aminoglycoside	AMI, TOB
42021121	46861231	1208	II 42:r:-	*aac(6')-Iaa*	Aminoglycoside	AMI, TOB
41761121	46641231	1208	II 42:r:-	*aac(6')-Iaa*	Aminoglycoside	AMI, TOB
41801121	46681237	639	Orion	*aac(6')-Iaa*	Aminoglycoside	AMI, TOB
41881121	46731231	1208	II 42:r:-	*aac(6')-Iaa*	Aminoglycoside	AMI, TOB
42291121	47051231	2014	Umbilo	*aac(6')-Iaa*	Aminoglycoside	AMI, TOB
43381121	47391231	473	Hadar	*aac(6')-Iaa,aph(3'')-Ib,aph(6)-Id,dfrA14,sul2,tet(A)*	Aminoglycoside, folate pathway antagonist, tetracycline	AMI, DOX, STR, S, TET, TOB, TMP
43511121	47451231	1208	II 42:r:-	*aac(6')-Iaa*	Aminoglycoside	AMI, TOB
43681121	43681471	19	Typhimurium	*aac(6')-Iaa*	Aminoglycoside	AMI, TOB
44021121	47521231	1208	II 42:r:-	*aac(6')-Iaa*	Aminoglycoside	AMI, TOB

*AMI, Amikacin; TOB, Tobramycin; DOX, Doxycycline; STR, Streptomycin; S, Sulfamethoxazole; TET, Tetracycline; TMP, Trimethoprim; CIP, Ciprofloxacin; GM, Gentamicin; NA, Nalidixic acid; SPT, Spectinomycin; FOS, Fosfomycin.
